# A phase I, single-center, randomized, open-label, three-period crossover study to evaluate the drug–drug interaction between ZSP1273 and oseltamivir in healthy Chinese subjects

**DOI:** 10.1128/aac.01729-24

**Published:** 2025-02-24

**Authors:** Yanqing Pang, Haijun Li, Xuemei Chen, Yingying Cao, Hui Jiang, Jufang Huang, Yiming Liu

**Affiliations:** 1 Department of Phase I Clinical Research Center, The Second Affiliated Hospital of Guangzhou University of Chinese Medicine (Guangdong Provincial Hospital of Chinese Medicine) 74715 , Guangzhou, Guangdong, China; 2 Department of Anatomy and Neurobiology, School of Basic Medical Sciences, Central South University 618101 , Changsha, Hunan, China; Providence Portland Medical Center, Portland, Oregon, USA

**Keywords:** drug-drug interaction, ZSP1273, oseltamivir, pharmacokinetics, safety

## Abstract

ZSP1273 is a novel small-molecule anti-influenza drug that targets the RNA polymerase PB2 subunit, while oseltamivir is the first-line medication that inhibits neuraminidase. ZSP1273 showed high efficacy against human influenza viruses both *in vitro* and *in vivo*, including oseltamivir-resistant strains *in vitro*. In future clinical applications, the combination of these two antiviral drugs with different mechanisms can reduce the potential for antiviral resistance that may arise from monotherapy. To evaluate the drug–drug interaction between ZSP1273 and oseltamivir by the pharmacokinetics and safety of co-administration in healthy subjects, a phase I, single-center, randomized, open-label, three-period crossover study was conducted. Thirty-six subjects enrolled were randomized in a 1:1:1 ratio into three crossover treatment sequences with oral administration detailed as follows: treatment A: ZSP1273 tablets 600 mg once daily (QD) for 5 days; treatment B: oseltamivir capsules 75 mg twice daily (BID) for 5 days; treatment C: ZSP1273 tablets 600 mg once daily (QD) + oseltamivir capsules 75 mg twice daily (BID) for 5 days. Plasma samples were collected from all subjects at scheduled time points after drug administration to measure the plasma concentrations of ZSP1273, oseltamivir, and its active metabolite oseltamivir carboxylate, for pharmacokinetic analysis. Compared with monotherapy, the geometric mean ratios (90% confidence intervals) of C_max,ss_, AUC_0-t,ss_, AUC_0-τ,ss_, and AUC_0-∞,ss_ for ZSP1273 after co-administration were all within the ineffective boundary range of 80% to 125%, supporting that no drug–drug interaction occurs with ZSP1273. After co-administration, the AUC_0-t,ss_, AUC_0-τ,ss_, and AUC_0-∞,ss_ of oseltamivir were all within 80% to 125%, while C_max,ss_ decreased by 39.9%. The pharmacokinetic parameters above of oseltamivir carboxylate remained within 80%–125%, except only the lower bound of the 90% CI for C_max,ss_ slightly below 80% (77.0%). Considering the rapid metabolism of oseltamivir into the active metabolite oseltamivir carboxylate and the minor impact of co-administration on the pharmacokinetic parameters of oseltamivir carboxylate, it is believed that no clinically significant drug–drug interaction was observed with the combination of these two drugs. During the trial, the safety and tolerability of both combination therapy and monotherapy were good, with no increased safety risks observed from the combination therapy.

**CLINICAL TRIALS:**

This study is registered with ClinicalTrials.gov as NCT05108051.

## INTRODUCTION

Influenza is a significant public health challenge due to its high morbidity and mortality rates (1). The influenza virus, an RNA virus, comprises four types: A, B, C, and D (2, 3), with influenza A infecting many species and being the primary cause of outbreaks and global pandemics (4). Currently, there are three main classes of anti-influenza drugs, differentiated by their mechanisms of action: M2 ion channel inhibitors, neuraminidase inhibitors, and RNA-dependent RNA polymerase inhibitors (5, 6). Neuraminidase inhibitors (oseltamivir, zanamivir, and peramivir) are the first-line treatments for influenza A (7). However, the emergence of drug resistance remains a concern (8, 9). Antiviral drugs targeting the RNA-dependent RNA polymerase subunits of influenza are considered the most promising (10). These subunits include the polymerase acidic protein, polymerase basic protein-1 (PB1), and polymerase basic protein-2 (PB2) (11). Baloxavir marboxil (S-033188), a polymerase acidic protein inhibitor, is a first-line medication for treating children and adults with uncomplicated influenza A and B infections. However, the increased use of this drug has led to the gradual emergence of baloxavir-resistant strains (12, 13). Pimodivir (JNJ-63623872, VX-787), a PB2 inhibitor, showed initial promise but was halted in a phase 3 trial due to lack of added benefit over current treatments (14–16). Its drug–drug interaction with oseltamivir was also assessed in a previous study, and no clinically relevant drug–drug interactions was observed (17). Currently, there are no clinically available anti-influenza drugs targeting the PB2 subunit. The rapid evolution of the influenza virus, resulting in reduced vaccine effectiveness and drug-resistant strains, underscores the need for continuous research into new anti-influenza medications.

ZSP1273 is a novel small-molecule drug that targets the RNA polymerase PB2 subunit by inhibiting the cap-snatching activity of the influenza polymerase complex. *In vitro* studies have demonstrated ZSP1273’s efficacy against human influenza A and avian influenza A(H7N9) viruses, including strains resistant to oseltamivir. *In vivo* studies in mice with acute influenza A (2009 H1N1) infection showed that ZSP1273 effectively reduced pulmonary viral loads and mortality compared with oseltamivir or pimodivir. An influenza virus cytopathic assay revealed that the synergy and antagonism indices of ZSP1273 combined with oseltamivir were 852.41 and −0.19, respectively, suggesting that these two drugs have strong synergistic effects (18). In a clinical phase I study evaluating different doses of ZSP1273 in healthy volunteers, no apparent safety concerns were noticed, even at doses up to 1,200 mg (19). In a clinical phase II study evaluating safety and efficacy of ZSP1273 in adults with acute uncomplicated influenza A infection, it showed a safety profile comparable to placebo, as well as higher efficacy than placebo in ameliorating influenza symptoms and lowering the viral load in adult patients, especially the 600 mg once per day (20). Even more, a clinical phase III study with a larger population (NCT04683406) is currently underway, to further evaluate ZSP1273’s efficacy against placebo and oseltamivir in adults with influenza infection. ZSP1273 is metabolized by glucuronosyltransferase, while oseltamivir is converted to oseltamivir carboxylate via carboxylesterases. The plasma protein binding rate of ZSP1273 in humans is greater than 98.9%, while the active metabolite of oseltamivir has a negligible plasma protein binding rate (approximately 3%) (21). ZSP1273 and its metabolites are primarily excreted in feces, while more than 99% of oseltamivir carboxylate is excreted by the kidneys (22). The known pharmacologic profiles of the drugs suggest that the potential for drug–drug interactions between ZSP1273 and oseltamivir is minimal.

Given the emergence of drug-resistant mutations in influenza A and the potent pharmacological effect of ZSP1273, including its good inhibitory activity against oseltamivir-resistant influenza virus strains and its highly synergistic effect when used in combination with oseltamivir, the combination of these two antiviral drugs with different mechanisms of action can reduce the potential for antiviral resistance that may arise from monotherapy. Therefore, this study aims to explore the drug–drug interactions between ZSP1273 with oseltamivir, to provide valuable information for combination therapy to reduce the occurrence of resistance, improve efficacy, and enhance the treatment of influenza.

## MATERIALS AND METHODS

The study was conducted at Guangdong Provincial Hospital of Chinese Medicine (Guangzhou, Guangdong, P.R. China) in accordance with the Declaration of Helsinki and the Good Clinical Practice guidelines of the National Medical Products Administration in China (23). The study was registered at ClinicalTrials.gov as NCT05108051.

### Participants

Eligible subjects were healthy males and females, aged 18 to 45 years with a body mass index (BMI) within the range of 18.0 to 26.0 kg/m^2^. Simultaneously, male subjects weighed at least 50 kg, and female subjects weighed at least 45 kg. Physical examination, vital signs, clinical laboratory tests, and electrocardiogram were normal, or any abnormalities were clinically insignificant.

The main exclusion criteria included individuals with allergies; history of swallowing difficulties or any gastrointestinal diseases affecting drug absorption; participation in any drug clinical trial or use of investigational drugs within 3 months prior to taking the study medication; diseases occurring within the 6 months prior to screening (including but not limited to gastrointestinal, renal, hepatic, neurological, hematological, endocrine, oncological, pulmonary, immunological, psychiatric, or cardiovascular diseases); and pregnancy.

### Study design

**Fig 1:**
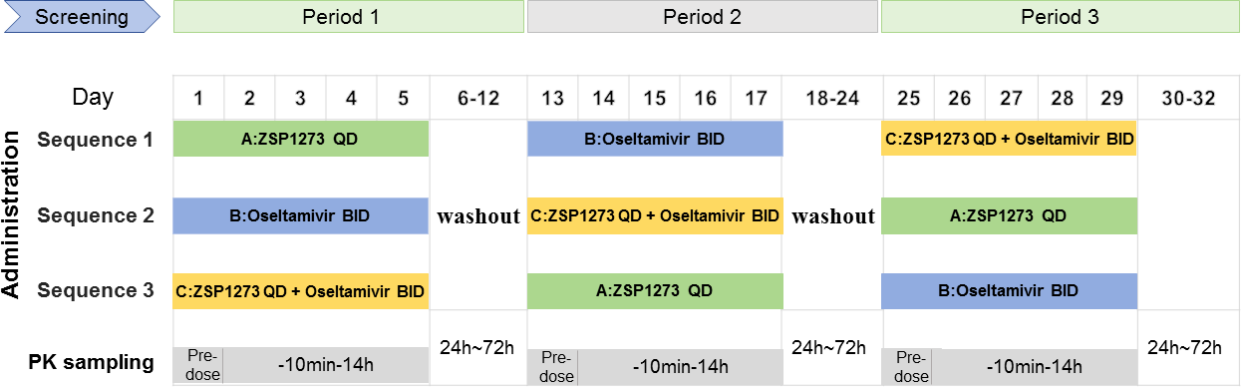
Study design. A single dose of oseltamivir capsule in the morning on the 5th day of each period.

The study design was summarized in Fig. 1. This study employed a single-center, randomized, open-label, three-period crossover design with 36 enrolled subjects to evaluate the pharmacokinetic interaction between ZSP1273 and oseltamivir. The 36 enrolled subjects were randomly assigned to one of three crossover treatment sequences in a 1:1:1 ratio. Each sequence consisted of three treatment periods: treatment A: ZSP1273 tablets 600 mg once daily (QD); treatment B: oseltamivir capsules 75 mg twice daily (BID); treatment C: ZSP1273 tablets 600 mg once daily (QD) + oseltamivir capsules 75 mg twice daily (BID). In each period, the subjects took the corresponding medication for five consecutive days according to their treatment sequence, with the medication administered only once in the morning on the 5th day of each cycle (i.e., a single dose of oseltamivir capsule on the 5th day). There was a 7-day washout period between each treatment period.

During days 1 to 4 of each period, subjects fasted for at least 10 h overnight, then took ZSP1273 or oseltamivir with 240 mL of warm water in the morning, followed by breakfast 2 h after administration. A second dose of oseltamivir was taken 2 h before dinner. On day 5, subjects fasted for at least 10 h overnight, refrained from drinking water for 1 h before dosing, then took ZSP1273 or oseltamivir with 240 mL of warm water in the morning, followed by continued water restriction for 2 h and fasting for 4 h after administration, with lunch eaten 4 h after dosing.

This study sets blood sampling (4 mL per sample) to be conducted within 1 h before drug administration on day 1 of each period, and intensive sampling on day 5 of each dosing period, including within 10 min before dosing, and at 15 min, 30 min, 45 min, 1 h, 1.5 h, 2 h, 2.5 h, 3 h, 4 h, 6 h, 8 h, 10 h, 12 h, 14 h, 24 h, 48 h, and 72 h post-dose.

### Pharmacokinetic analysis

A liquid chromatography–tandem mass spectrometry (LC–MS/MS) method was used to determine the concentrations of ZSP1273, oseltamivir, and oseltamivir carboxylate in plasma. The analytical method had undergone rigorous method validation (24). ZSP1273-d_4_, oseltamivir-d_3_, and oseltamivir carboxylate-d_3_ were used as stable isotope-labeled internal standards for ZSP1273, oseltamivir, and oseltamivir carboxylate, respectively. Separation was done on an ultimate XB C18 column, and detection was carried out on a triple quad 4,500 multiple reaction monitoring (MRM) transitions 441.2→289.2 and 445.2→293.0 for ZSP1273 and internal standard, respectively; 313.1→208.1 and 316.1→211.1 for oseltamivir and internal standard, respectively; 285.1→138.1 and 288.1→138.1 for oseltamivir carboxylate and internal standard, respectively (25–28). The standard curve concentration range of ZSP1273 was 10.0–10,000 ng/mL, oseltamivir was 0.500–200 ng/mL, oseltamivir carboxylate was 4.00–500 ng/mL, respectively.

The mean (standard deviation) plasma concentration–time profile of ZSP1273, oseltamivir, and oseltamivir carboxylate was graphically presented. Pharmacokinetic parameters were calculated using a non-compartmental model based on individual blood drug concentrations and actual sampling times for each subject. WinNonlin 8.0 was used for the determination or calculation of pharmacokinetic parameters. The major pharmacokinetic parameters included maximum plasma concentration (C_max,ss_), time to C_max,ss_ (T_max,ss_), area under plasma concentration–time curve from 0 to t (AUC_0-t,ss_), area under the plasma concentration–time curve for the defined interval between doses (AUC_0-τ,ss_), and area under the plasma concentration–time curve from 0 to infinity (AUC_0-∞,ss_).

### Safety assessment

Safety assessment was conducted from the first administration of the investigational drug until the end of the trial. Safety indicators included adverse events (AEs), serious adverse events (SAEs), clinical laboratory tests (hematology, biochemistry, and urinalysis), vital signs, physical examination, and 12-lead electrocardiogram.

The severity of AE was assessed according to the CTCAE 5.0 standard and includes grades 1 to 5. Based on the criteria for determining causality between drugs and adverse events, the relationship was classified into five levels: definitely related, probably related, possibly related, probably not related, definitely not related. Among them, definitely related, probably related, and possibly related are defined as drug-related AEs.

### Statistical analysis

Statistical analysis was performed using SAS version 9.4. The extent of drug–drug interaction (DDI) was assessed by calculating the geometric mean ratios (GMRs) of the drug administered in combination versus alone, along with the corresponding 90% confidence intervals (*CIs*) based on the log-transformed data. The no effect boundary was considered to be the 90% *CIs* of the GMRs for the pharmacokinetic parameters that fell within the range of 80%–125%, concluding that there is no drug–drug interaction between the two drugs. When analyzing pharmacokinetic parameters, if the area under the plasma concentration–time curve extrapolated to infinity (AUC__%Extrap_) of subjects was >20.0% or the Rsq was <0.800, the AUC_0-∞,ss_ of these subjects was unreliable, and thus they were excluded from further analysis (29).

Adverse events were coded using Med DRA 23.1 Chinese (30) and analyzed based on system organ class (SOC) and preferred term (PT). Adverse events during the treatment period, drug-related adverse events, and serious adverse events were described by frequency, number of cases (n), and incidence rate (%) according to SOC, PT, and the highest severity classification.

## RESULTS

### Participants

A total of 36 subjects were enrolled in this trial, including 24 males and 12 females, with an average age of 29.4 ± 6.5 (range: 19 to 43) years, average height of 162.9 ± 9.2 (range: 148.5 to 186.5) cm, average weight of 59.8 ± 7.6 (range: 46.9 to 79.8) kg, and average BMI of 22.5 ± 1.6 (range: 18.5 to 25.6) kg/m^2^. The age, weight, and BMI were compliant with the eligible criteria.

All of subjects received the study drug at least once. But during the trial, a total of three subjects withdrew from the study in a different period. As the result, there were 36 subjects who received ZSP1273; 34 subjects who received oseltamivir; and 34 subjects who received ZSP1273 + oseltamivir. All of them were included in the pharmacokinetic analysis.

### Pharmacokinetic study results

**Fig 2:**
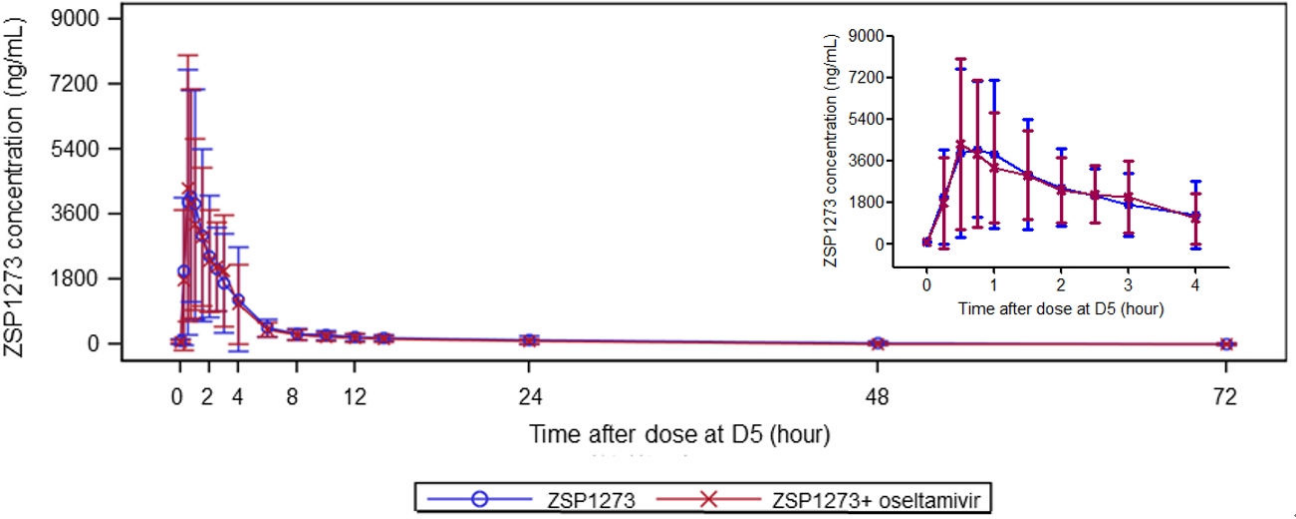
Mean plasma concentration–time curve of ZSP1273.

**Fig 3:**
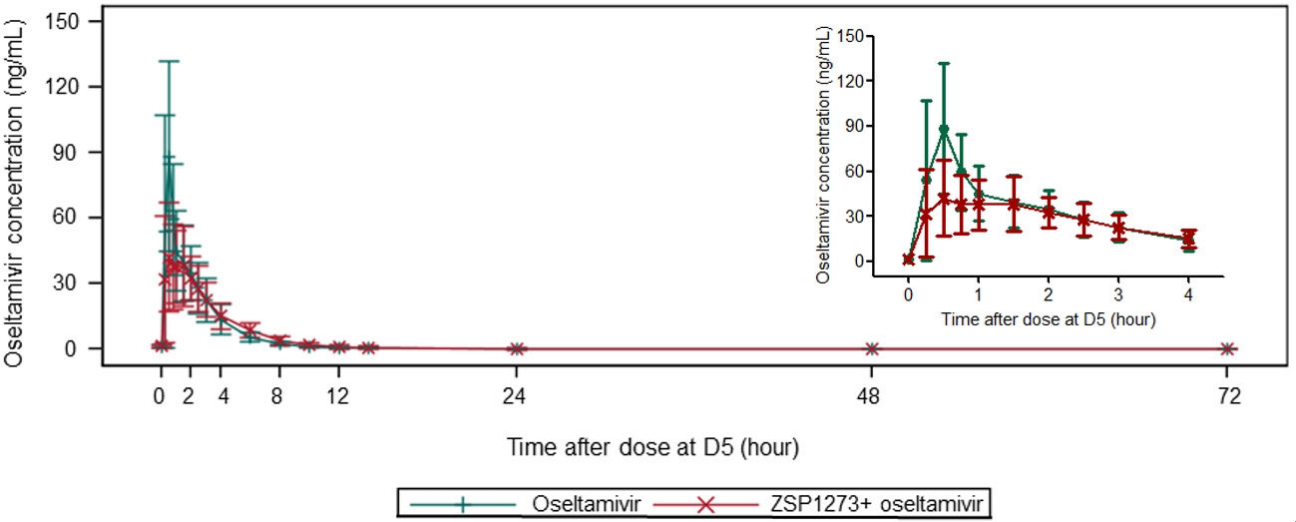
Mean plasma concentration–time curve of oseltamivir.

**Fig 4:**
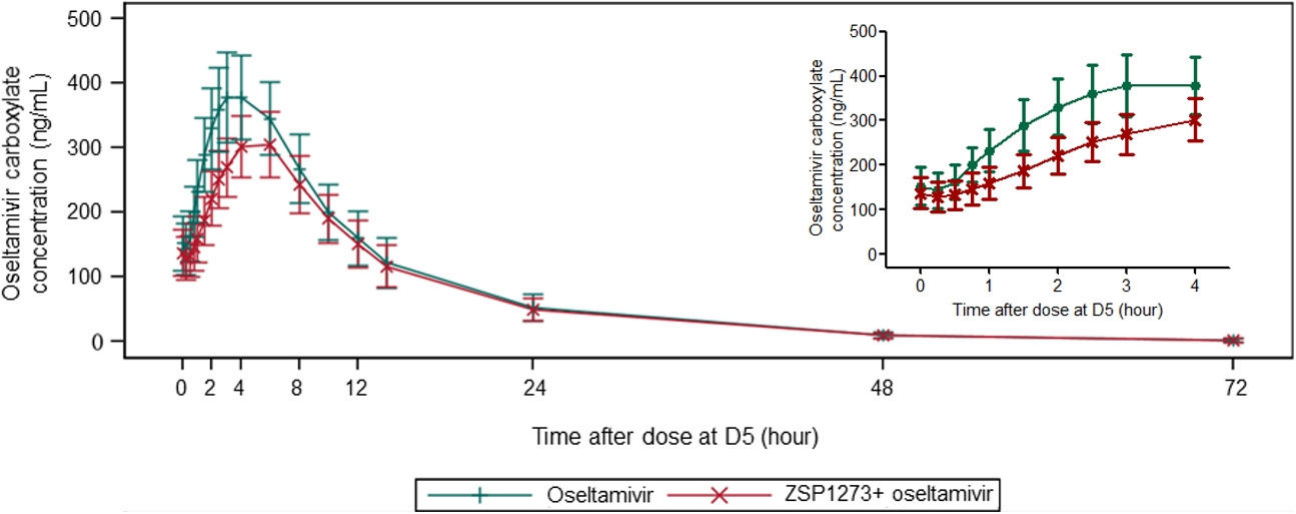
Mean plasma concentration–time curve of oseltamivir carboxylate.

The plasma concentration–time curves of ZSP1273, oseltamivir, and oseltamivir carboxylate in plasma are presented in Fig. 2 to 4. The pharmacokinetic parameters are detailed in Table 1. The geometric mean ratios (GMRs) (90% *CIs*) for the major pharmacokinetic parameters C_max,ss_, AUC_0-t,ss_, AUC_0-τ,ss_, and AUC_0-∞,ss_ of ZSP1273, oseltamivir, and oseltamivir carboxylate were calculated. For ZSP1273, there was no apparent change in exposure when co-administration with oseltamivir with GMRs (90% *CIs*) wholly contained within the 80%–125% no-effect boundaries (C_max,ss_: 99.9% [82.5%–120.9%]; AUC_0-t,ss_: 92.8% [85.6%–100.7%]; AUC_0-τ,ss_: 96.3% [89.2%–103.8%]; AUC_0-∞,ss_: 91.8% [83.4%–100.9%]), supporting the absence of drug–drug interaction with ZSP1273 (Table 2). For oseltamivir, except for a 39.9% decrease in C_max,ss_ (91.2→55.3 ng/mL), all other parameter’s GMRs (90% *CIs*) were within the range of 80%–125% (AUC_0-t,ss_: 92.7% [88.3%–97.3%]; AUC_0-τ,ss_: 92.2% [88.2%–96.5%]; AUC_0-∞,ss_: 92.6% [88.2%–97.3%]) (Table 3). For oseltamivir carboxylate, except for the lower limit of the 90% *CI* of C_max,ss_ was slightly below 80% (77.0%), all other parameter’s GMRs (90% *CIs*) were within the range of 80%–125% (C_max,ss_: 80.4% [77.0%–84.0%]; AUC_0-t,ss_: 86.7% [83.7%–89.9%]; AUC_0-τ,ss_: 83.3% [80.7%–85.9%]; AUC_0-∞,ss_: 87.1% [84.1%–90.2%]) (Table 4). Considering the rapid metabolism of oseltamivir into the active metabolite oseltamivir carboxylate in the body and the minimal impact on the pharmacokinetic parameters of oseltamivir carboxylate of co-administration, it is concluded that there is no clinically significant drug–drug interaction between the two drugs when used in combination.

**TABLE 1:** Pharmacokinetic parameters of ZSP1273, oseltamivir, and oseltamivir carboxylate in plasma after oral administration

PK parameters (unit)	Arithmetic mean ± SD (CV%)					
	ZSP1273 (*N* = 36)	Oseltamivir (*N* = 34)		ZSP1273 +oseltamivir (*N* = 34)		
	ZSP1273	Oseltamivir	Oseltamivir carboxylate	ZSP1273	Oseltamivir	Oseltamivir carboxylate
C_max,ss_ (ng/mL)	6,421.7 ± 3,660.6 (57.0%)	101.2 ± 49.2 (48.6%)	397.4 ± 64.4 (16.2%)	6,270.0 ± 3,369.7 (53.7%)	59.4 ± 24.5 (41.2%)	318.3 ± 49.2 (15.5%)
AUC_0-t,ss_ (h*ng/mL)	16,529.6 ± 7,808.2 (47.2%)	174.6 ± 39.0 (22.3%)	5177.5 ± 1038.2 (20.1%)	14,939.0 ± 6,027.0 (40.3%)	162.0 ± 35.1 (21.7%)	4,483.2 ± 955.0 (21.3%)
AUC_0-τ,ss_ (h*ng/mL)	14,487.1 ± 5,519.8 (38.1%)	171.6 ± 38.8 (22.6%)	3348.8 ± 472.6 (14.1%)	13,656.5 ± 4,758.0 (34.8%)	158.8 ± 35.3 (22.2%)	2,784.0 ± 427.1 (15.3%)
AUC_0-∞,ss_ (h*ng/mL)	18,392.1 ± 12,058.8 (65.6%)	179.4 ± 38.8 (21.6%)	5271.0 ± 1047.1 (19.9%)	15,865.8 ± 6,635.4 (41.8%)	166.3 ± 35.8 (21.6%)	4,583.0 ± 974.5 (21.3%)
T_max,ss_^*a*^ (h)	0.88 (0.25, 4.01)	0.50 (0.25, 2.50)	4.00 (2.50, 6.00)	1.50 (0.25, 4.00)	0.75 (0.25, 2.50)	5.00 (2.50, 6.01)
t_1/2,ss_ (h)	17.8 ± 33.6 (189.1%)	4.5 ± 4.8 (107.0%)	10.3 ± 1.6 (15.8%)	14.0 ± 10.3 (73.5%)	3.2 ± 2.8 (87.3%)	9.8 ± 1.6 (15.9%)
λ_z_ (1 /h)	0.074 ± 0.046 (62.5%)	0.280 ± 0.150 (53.6%)	0.069 ± 0.009 (13.5%)	0.070 ± 0.041 (58.7%)	0.318 ± 0.155 (48.7%)	0.072 ± 0.011 (14.7%)
CL_ss_/F (L/h)	47.4 ± 17.8 (37.5%)	459.8 ± 106.3 (23.1%)		48.6 ± 15.3 (31.4%)	495.5 ± 110.5 (22.3%)	
V_z_/F (L)	1,006.5 ± 1,265.7 (125.8%)	3,069.2 ± 3,494.1 (113.8%)		923.8 ± 659.5 (71.4%)	2,341.0 ± 2,282.7 (97.5%)	
C_trough_ (ng/mL)	86.3 ± 77.4 (89.6%)	1.3 ± 0.6 (48.5%)	152.1 ± 42.4 (27.9%)	77.7 ± 49.7 (64.0%)	1.4 ± 0.8 (56.4%)	137.7 ± 36.0 (26.2%)

^*a*^ T_max_ is expressed as the median (minimum, maximum). For oseltamivir carboxylate, CL_ss/F_ and V_z_/F are not calculated.

**TABLE 2:** Pharmacokinetic parameters analysis of ZSP1273 monotherapy and co-administration

PK parameters (unit)	Geometric mean		Geometric mean ratios		Intra-individual coefficient of variation (%)
	ZSP1273 monotherapy (*N* = 36)	ZSP1273 co- administration (*N* = 34)	Co-administration/monotherapy (%)	90% CI (%)	
C_max,ss_ (ng/mL)	5,517.1	5,466.0	99.9	(82.5,120.9)	49.2
AUC_0-t,ss_ (h*ng/mL)	15,031.4	13,926.7	92.8	(85.6,100.7)	19.9
AUC_0-τ,ss_ (h*ng/mL)	13,543.4	12,964.7	96.3	(89.2,103.8)	18.6
AUC_0-∞,ss_ (h*ng/mL)	16,036.9	14,749.3	91.8	(83.4,100.9)	23.4
AUC_0-∞,ss_ (h*ng/mL)	15,576.5^*a*^	14,219.8^*b*^	94.5	(86.7,103.0)	18.8

^*a*^ *N* = 33, three subjects were excluded from analysis because the AUC_%_Extrap_ of one subject was >20.0%, and the Rsq of two subjects was <0.800.

^*b*^ *N* = 29, five subjects were excluded from analysis because the AUC_%_Extrap_ of one subject was >20.0%, and the Rsq of four subjects was <0.800.

**TABLE 3:** Pharmacokinetic parameters analysis of oseltamivir monotherapy and co-administration

PK parameters (unit)	Geometric mean		Geometric mean ratios		Intra-individual coefficient of variation (%)
	Oseltamivir monotherapy (*N* = 34)	Oseltamivir co-administration (*N* = 34)	Co-administration/monotherapy (%)	90% CI (%)	
C_max,ss_ (ng/mL)	91.2	55.3	60.1	(51.9,69.7)	36.7
AUC_0-t,ss_ (h*ng/mL)	170.4	158.4	92.7	(88.3,97.3)	11.7
AUC_0-τ,ss_ (h*ng/mL)	167.3	155.0	92.2	(88.2,96.5)	10.8
AUC_0-∞,ss_ (h*ng/mL)	175.3	162.6	92.6	(88.2,97.3)	11.8
AUC_0-∞,ss_ (h*ng/mL)	175.1^*a*^	162.6	93.5	(89.4,97.8)	10.7

^*a*^ *N* = 33, one subject was excluded from analysis because the Rsq was <0.800.

**TABLE 4:** Pharmacokinetic parameters analysis of oseltamivir carboxylate monotherapy and co-administration

PK parameters (unit)	Geometric mean		Geometric mean ratios		Intra-individual coefficient of variation (%)
	Oseltamivir monotherapy (*N* = 34)	Oseltamivir co-administration (*N* = 34)	Co-administration/monotherapy (%)	90% CI (%)	
C_max,ss_ (ng/mL)	392.7	314.6	80.4	(77.0,84.0)	10.5
AUC_0-t,ss_ (h*ng/mL)	5,076.4	4,387.4	86.7	(83.7,89.9)	8.6
AUC_0-τ,ss_ (h*ng/mL)	3,315.4	2,751.8	83.3	(80.7,85.9)	7.5
AUC_0-∞,ss_ (h*ng/mL)	5,170.4	4,485.8	87.1	(84.1,90.2)	8.4

In ZSP1273 monotherapy, the percentage of the area under the plasma concentration–time curve extrapolated to infinity (AUC__%Extrap_) of one subject was >20.0%, and the Rsq of two subjects was <0.800. In oseltamivir monotherapy, the Rsq of one subject was <0.800. In ZSP1273 + oseltamivir combination therapy, the AUC__%Extrap_ of one subject was >20.0%, and the Rsq of four subjects was <0.800 when analyzing ZSP1273. It was deemed that the AUC_0-∞,ss_ of these subjects was unreliable, and thus they were excluded from further analysis. The results indicated that the GMRs (90% *CIs*) for AUC_0-∞,ss_ of ZSP1273 and oseltamivir were within the range of 80%–125% in combination therapy and monotherapy (Tables 2 and 3), consistent with the analysis results including all subjects.

### Safety assessment

A total of 36 healthy subjects were enrolled and administered the study drugs. After drug administration, adverse events occurred in 22 subjects (61.1%), including 19 subjects (52.8%) with drug-related adverse events. These included 11 subjects (11/36, 30.6%) receiving ZSP1273, 5 subjects (5/34,14.7%) receiving oseltamivir, and 7 subjects (7/34,20.6%) receiving ZSP1273 + oseltamivir. There were no serious adverse events, no adverse events leading to discontinuation of treatment, and no fatalities.

Common adverse events (cumulative incidence >15%) included diarrhea (13.9% [5/36] after ZSP1273 administration and 8.8% [3/34] after ZSP1273 + oseltamivir co-administration) and increased blood triglycerides (11.1% [4/36] after ZSP1273 administration, 2.9% [1/34] after oseltamivir administration, and 5.9% [2/34] after ZSP1273 + oseltamivir co-administration), all of which were drug-related adverse events. Compared with ZSP1273 or oseltamivir alone, the incidence and severity of adverse events did not increase with ZSP1273 + oseltamivir combination therapy. All drug-related AEs were of grade 1 severity. The incidence of adverse events by system organ class (SOC) and preferred term (PT) is detailed in Table 5.

**TABLE 5:** Incidence of AEs by system organ class (SOC) and preferred term (PT)

System organ class (SOC) Preferred term (PT)	Relationship to drugs	ZSP1273 (*N* = 36)		Oseltamivir (*N* = 34)		ZSP1273 +oseltamivir (*N* = 34)		Total (*N* = 36)	
		Cases (%)	Occurrences	Cases (%)	Occurrences	Cases (%)	Occurrences	Cases (%)	Occurrences
At least one treatment-emergent adverse event (TEAE)		12 (33.3)	24	7 (20.6)	14	7 (20.6)	8	22 (61.1)	46
At least one treatment-emergent adverse event related to drugs		11 (30.6)	22	5 (14.7)	6	7 (20.6)	8	19 (52.8)	36
Investigations		8 (22.2)	18	6 (17.6)	12	3 (8.8)	4	17 (47.2)	34
Increased blood triglycerides	Possibly related	4 (11.1)	4	1 (2.9)	1	2 (5.9)	2	7 (19.4)	7
Decreased hemoglobin	Probably not related	2 (5.6)	2	2 (5.9)	2	0	0	4 (11.1)	4
Decreased high-density lipoprotein (HDL)	Possibly related	1 (2.8)	1	2 (5.9)	2	1 (2.9)	1	4 (11.1)	4
Increased blood uric acid	Possibly related	2 (5.6)	2	1 (2.9)	1	0	0	3 (8.3)	3
Increased blood creatinine	Possibly related	3 (8.3)	3	0	0	0	0	3 (8.3)	3
Increased low-density lipoprotein (LDL)	Possibly related	2 (5.6)	2	0	0	0	0	2 (5.6)	2
Increased blood cholesterol	Possibly related	2 (5.6)	2	0	0	0	0	2 (5.6)	2
Increased gamma-glutamyl transferase (GGT)	Possibly related	1 (2.8)	1	0	0	0	0	1 (2.8)	1
Decreased neutrophil count	Possibly related	0	0	0	0	1 (2.9)	1	1 (2.8)	1
Nitrites present in urine	Probably not related	0	0	1 (2.9)	1	0	0	1 (2.8)	1
Crystals present in urine	Probably not related	0	0	1 (2.9)	1	0	0	1 (2.8)	1
Increased urine specific gravity	Probably not related	0	0	1 (2.9)	1	0	0	1 (2.8)	1
Positive urine occult blood	Probably not related	0	0	1 (2.9)	1	0	0	1 (2.8)	1
Protein detected in urine	Probably not related	0	0	1 (2.9)	1	0	0	1 (2.8)	1
Increased lipase	Possibly related	1 (2.8)	1	0	0	0	0	1 (2.8)	1
Decreased blood triglycerides	Possibly related	0	0	1 (2.9)	1	0	0	1 (2.8)	1
Gastrointestinal disorders		5 (13.9)	5	0	0	3 (8.8)	3	7 (19.4)	8
Diarrhea	Probably related	5 (13.9)	5	0	0	3 (8.8)	3	7 (19.4)	8
Skin and subcutaneous tissue disorders		1 (2.8)	1	1 (2.9)	1	0	0	2 (5.6)	2
Maculopapular rash	Possibly related	1 (2.8)	1	0	0	0	0	1 (2.8)	1
Acneiform dermatitis	Possibly related	0	0	1 (2.9)	1	0	0	1 (2.8)	1
General disorders and administration site conditions		0	0	0	0	1 (2.9)	1	1 (2.8)	1
Fever	Possibly related	0	0	0	0	1 (2.9)	1	1 (2.8)	1
Infections and infestations		0	0	1 (2.9)	1	0	0	1 (2.8)	1
Upper respiratory tract infection (URTI)	Probably not related	0	0	1 (2.9)	1	0	0	1 (2.8)	1

In summary, the safety profile of ZSP1273 administered alone or in combination with oseltamivir in healthy subjects was favorable.

## DISCUSSION

This study employed a single-center, randomized, open-label, three-period crossover design to explore the drug–drug interaction of combination of influenza antiviral drugs with different mechanisms of action to provide supplement to circumvent the challenge of antiviral resistance that may arise with monotherapy.

The results of this study indicated that after achieving steady-state with co-administration and monotherapy, the GMRs of C_max,ss_ and AUC for ZSP1273 and oseltamivir carboxylate, and the GMR of AUC for oseltamivir were between 80.4% and 99.9%. There were no significant changes in the pharmacokinetic parameters of either component in either dosing regimen. The 90% *CIs* of C_max,ss_ and AUC for ZSP1273 were within the range of 80% to 125%, indicating that co-administration of the two drugs did not affect the pharmacokinetics of ZSP1273, and no dose adjustment of ZSP1273 is necessary for clinical use. The GMRs (90% *CIs*) of the AUC for oseltamivir and oseltamivir carboxylate were within the 80%–125% range. The C_max,ss_ of oseltamivir decreased by 39.9%. Considering that oseltamivir is rapidly metabolized into the active metabolite oseltamivir carboxylate, this change in peak concentration is of no clinical significance. For oseltamivir carboxylate, the lower limit of the 90% CI for the GMR of C_max,ss_ (77.0%) was slightly below 80%, which can be attributed to variability as C_max_ is a single-point measurement. Preclinical studies suggest that the potential for drug–drug interactions between ZSP1273 and oseltamivir is minimal. Oseltamivir is almost completely converted into the active metabolite oseltamivir carboxylate by carboxylesterases, while ZSP1273 is primarily metabolized by glucuronosyltransferase. Absorbed oseltamivir is mainly eliminated by conversion to oseltamivir carboxylate, with more than 99% of oseltamivir carboxylate being excreted by the kidneys. ZSP1273 and its metabolites are primarily excreted in feces. In this study, oseltamivir did not affect the PK parameters of ZSP1273, and there was no significant change in the AUC of oseltamivir when co-administered. Therefore, the slight decrease in C_max_ of the active metabolite oseltamivir carboxylate is of no clinical significance. In conclusion, there was no clinically significant drug–drug interaction between the two drugs, and no dose adjustment was necessary for clinical use. There were some GMR values closer to the lower boundary of 80%–125%, albeit within boundary. These instances may appear to indicate subtle changes in drug exposure when the two drugs were co-administered. Several factors could contribute to these, such as age, gender, gastrointestinal motility, and other individual variability factors. These deviations were small and did not result in clinically significant drug–drug interactions. They may be noted in future clinical trials and actual clinical practice.

Among the 36 participants enrolled in the study and administered the investigational drug, 22 participants (61.1%) experienced adverse events, mainly abnormalities detected through laboratory tests after withdrawal. Drug-related adverse events occurred in 19 participants (52.8%). Adverse events with a combined incidence of over 15% included diarrhea (ZSP1273 [5/36, 13.9%], ZSP1273 + oseltamivir [3/34, 8.8%]) and increased blood triglycerides (ZSP1273 [4/36, 11.1%], oseltamivir [1/34, 2.9%], and ZSP1273 + oseltamivir [2/34, 5.9%]), which were also classified as drug-related adverse events. Compared with ZSP1273 or oseltamivir monotherapy, the incidence and severity of adverse events did not increase with the combined administration of ZSP1273 + oseltamivir. All drug-related AEs were of grade 1 severity. There were no serious adverse events and no fatalities. The safety profile of monotherapy or co-administered was favorable. Conducting an open-label trial with an experimental and a known drug may introduce potential bias in reporting treatment-emergent adverse event (TEAE). Investigators may unconsciously or consciously report adverse events differently based on their expectations of the drug’s safety profile when knowing which drug the participant received. To address this concern, we implemented several measures to mitigate bias, such as employing objective criteria, including clinical laboratory tests, vital signs, physical examination, and 12-lead electrocardiogram for assessing TEAE and utilizing the standardized reporting tool Med DRA 23.1 for consistent classification of adverse events. Additionally, investigators and staff were thoroughly trained to minimize subjective influences during data collection and reporting.

In summary, the findings of this study indicated that the concurrent administration of ZSP1273 and oseltamivir was associated with a favorable safety profile and high tolerability. Notably, no clinically relevant drug–drug interactions (DDIs) were identified during the co-administration of ZSP1273 with oseltamivir. The absence of interactions means that developers can more easily predict the efficacy and safety of combination therapies, reducing the complexity and uncertainty often associated with drug interactions. This can shorten the clinical trial timeline and lower development costs, allowing new drugs to reach the market faster to meet patient needs. For clinical application, the lack of interaction offers significant advantages for clinical treatment. Physicians can devise more effective combination therapy regimens without the concern of drug interactions. This safety enhances patient adherence and reduces the risk of adverse effects, ensuring that patients achieve the best therapeutic outcomes. Compared with drug combinations with interactions, those without interactions provide greater flexibility and safety for clinical practice. These results provided valuable insights for guiding the judicious utilization of this combination therapy in both clinical trial protocols and real-world clinical practice.
